# Elevated Expression of Stromal Palladin Predicts Poor Clinical Outcome in Renal Cell Carcinoma

**DOI:** 10.1371/journal.pone.0021494

**Published:** 2011-06-28

**Authors:** Vivekanand Gupta, Daniel E. Bassi, Jeffrey D. Simons, Karthik Devarajan, Tahseen Al-Saleem, Robert G. Uzzo, Edna Cukierman

**Affiliations:** 1 Cancer Biology Program, Fox Chase Cancer Center, Philadelphia, Pennsylvania, United States of America; 2 Department of Pathology, Fox Chase Cancer Center, Philadelphia, Pennsylvania, United States of America; 3 Department of Surgical Oncology, Fox Chase Cancer Center, Philadelphia, Pennsylvania, United States of America; 4 Department of Biostatistics, Fox Chase Cancer Center, Philadelphia, Pennsylvania, United States of America; Deutsches Krebsforschungszentrum, Germany

## Abstract

The role that stromal renal cell carcinoma (RCC) plays in support of tumor progression is unclear. Here we sought to determine the predictive value on patient survival of several markers of stromal activation and the feasibility of a fibroblast-derived extracellular matrix (ECM) based three-dimensional (3D) culture stemming from clinical specimens to recapitulate stromal behavior *in vitro*. The clinical relevance of selected stromal markers was assessed using a well annotated tumor microarray where stromal-marker levels of expression were evaluated and compared to patient outcomes. Also, an *in vitro* 3D system derived from fibroblasts harvested from patient matched normal kidney, primary RCC and metastatic tumors was employed to evaluate levels and localizations of known stromal markers such as the actin binding proteins palladin, alpha-smooth muscle actin (α-SMA), fibronectin and its spliced form EDA. Results suggested that RCCs exhibiting high levels of stromal palladin correlate with a poor prognosis, as demonstrated by overall survival time. Conversely, cases of RCCs where stroma presents low levels of palladin expression indicate increased survival times and, hence, better outcomes. Fibroblast-derived 3D cultures, which facilitate the categorization of stromal RCCs into discrete progressive stromal stages, also show increased levels of expression and stress fiber localization of α-SMA and palladin, as well as topographical organization of fibronectin and its splice variant EDA. These observations are concordant with expression levels of these markers *in vivo.* The study proposes that palladin constitutes a useful marker of poor prognosis in non-metastatic RCCs, while *in vitro* 3D cultures accurately represent the specific patient's tumor-associated stromal compartment. Our observations support the belief that stromal palladin assessments have clinical relevance thus validating the use of these 3D cultures to study both progressive RCC-associated stroma and stroma-dependent mechanisms affecting tumorigenesis. The clinical value of assessing RCC stromal activation merits further study.

## Introduction

In the United States, about 58,240 new renal cancers and 13,040 related deaths took place during 2010 [1]. Metastasis to distant sites, especially lung, bone, brain and liver, account for the majority of the morbidity associated with Renal Cell Carcinoma (RCC) [2]. Because surgery and available targeted therapies have limited impact on survival in patients with advanced RCC, it is believed that alternative approaches, such as targeting the primary and/or secondary tumor microenvironment, could improve clinical outcomes. A characteristic of renal cancers is that it contains a fibrous-like stromal reaction that directly intercalates with the cancerous epithelia [3]. It is well accepted that tumor-associated stroma, including activated or desmoplastic stromal fibroblasts known as tumor- or cancer-associated fibroblasts and their self derived extracellular matrix (ECM), play a major role in cancer development, progression, invasion and metastasis [4], [5], [6]. In order to test if kidney stroma and its tumor-activated (or tumor-associated) ECM play pivotal roles in renal tumorigenesis, we screened a cohort of RCC and normal histological human kidney samples and determined the clinical significance of scoring stromal activation -where increased collagen as well as myofibroblastic features were positively identified- for prognostic purposes. Prompted by results stemming from this screen, a small sample of human fibroblasts were harvested from fresh surgical tissues obtained from primary renal tumors, patient-paired normal adjacent kidney tissue, and from metastatic sites. We used a fibroblast-derived ECM based three-dimensional (3D) system of high physiological relevance, which effectively mimics the *in vivo* stroma onset of various epithelial tumors [7], [8], [9], to stage the stroma into discrete and well characterized levels of activation or stromal progression as a novel means to classify RCC and compared it with the established RCC staging (e.g., TMN classification) systems. The Fuhrman Classification system used to evaluated RCC progression using TMN classification has been regarded as an imperfect system that does not always predict the clinical outcome for individuals at risk [10]. Furthermore, despite the successful identification of selected epithelial marker proteins that characterize the stages of epithelial transformation [11], none of these has been shown to serve as useful prospective markers. We have previously demonstrated that *in vitro* stromal sorting can be accomplished by assessing several aspects of cell-derived 3D cultures such as ECM organization and expression of known tumor-stroma markers such as alpha-smooth muscle actin (α-SMA) [7], [8], [9]. To better assess RCC-associated stromal progression, here we investigate additional stromal markers which were simultaneously assessed *in vivo* using the same original samples: Palladin, an early myofibroblast differentiation marker localized to fibroblastic stress fibers [12]; EDA, a spliced form of fibronectin, upregulated during renal tumor stromal activation [3]; and uPARAP/Endo180, also upregulated in the activated tumor microenvironment [13] and tentatively associated to ECM (e.g., collagen I) degradation [14], facilitating tumor invasion and metastasis [15].

## Results

### Stromal palladin is a predictor of poor prognosis in renal cell carcinoma

**Figure 1:**
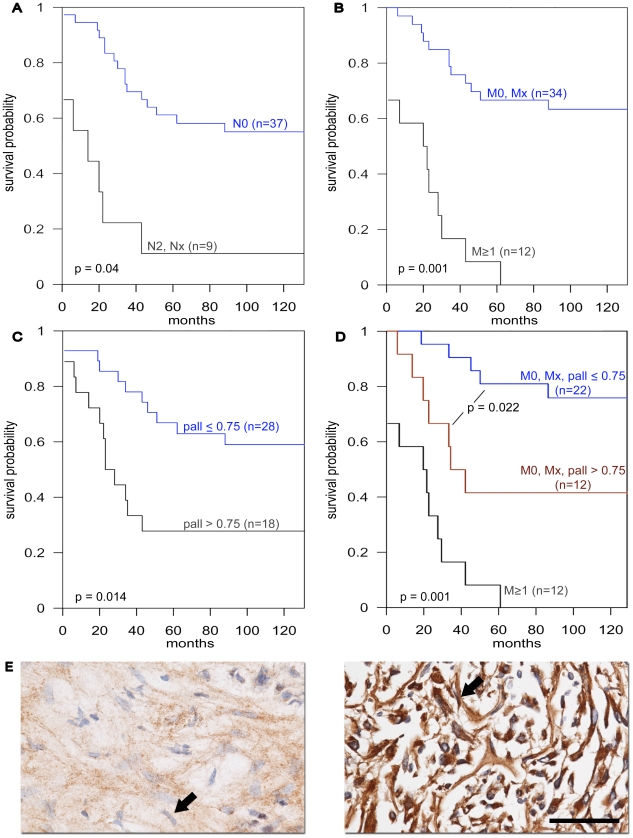
RCC distributions and stromal palladin expression levels as they relate to patient outcomes. The depicted graphs (**A-D**) correspond to Kaplan-Meier curves where the x-axis denotes the time-scale (in months) and the y-axis denotes the corresponding survival probability (e.g., survival fraction). **A** separates the group of cases identified as node positive (N2, Nx) from the node negative (N0) ones. **B** sorts cases by metastatic positive (M1) vs. negative (M0, Mx). **C** separates the tumor cohort by low (palladin ≤0.75) and high (palladin >0.75) stromal palladin expressions and **D** multivariate CART-based sorting of the cohort where non metastatic patients (M0, Mx) were further sorted by their stromal palladin expression levels (palladin ≤0.75 or >0.75). The corresponding p values are provided. **E** are representative immunohistochemistry images of palladin staining showing low (left panel) and high (right panel) examples or expression levels. Arrows point to the types of scored fibroblastic cells that rendered the stromal palladin scores. Barr represents 100 µM. Note how high levels of stromal palladin are indicative of smaller survival probabilities.

In order to determine the clinical relevance of α-SMA, palladin, uPARAP/Endo 180 and EDA in RCCs, and hence their possible usefulness as prospective markers, we analyzed their levels of expression in the stroma of 53 normal and RCC surgical samples that constituted a tumor microarray (TMA) described in Table 1. First, we tested whether our TMA was representative of known human RCC occurrences by analyzing links between node positive or metastatic RCC instances and survival rates. Univariate analyses using CART revealed that survival time was significantly decreased in node positive compared to node negative (p-value  = 0.004, Figure 1A) and metastatic vs. non-metastatic cases (p-value  = 0.001, Figure 1B). Next, immunohistological scored evaluations consisting of 0, 0.5, 1, or 2 values were assigned to each sample by a blinded pathologist who was instructed to only score stromal levels of expression for each of the assorted markers (see examples for palladin in Figure 1e). The correlation between immunohistological staining and clinical parameters such as presence of local or distant metastases and survival was analyzed. Among all the markers tested, the expression of palladin in stromal fibroblasts showed the strongest correlation with overall survival. Patients whose stromal palladin staining scores were at or below 0.75 showed a significantly higher survival than those with a higher level (p-value  = 0.014, Figure 1c). Importantly, the same correlation was observed in the subpopulation of patients that did not present metastatic onsets at the time of their surgical procedure. Figure 1d shows how using multivariable CART analysis non-metastatic patients whose palladin levels were 0.75 or below were found to have significantly improved survival compared to those with higher palladin levels (>0.75) (p-value = 0.022). Only 8% of bona fide metastatic patients (M1) were found to be alive at 60 months while 40% of non-metastatic patients with a palladin level exceeding 0.75 and 80% of non-metastatic patients with a palladin level at or below 0.75 were found to survive 60 months. These results indicate that non-metastatic patients with lower stromal palladin levels (≤0.75) survive the longest compared to other groups. Correlation between α-SMA and palladin levels was observed to be 0.58 (95% CI (0.36, 0.73) suggesting a similar stromal distribution for these two markers. Although α-SMA stromal expression levels presented a similar distribution to the one observed for palladin, the background expression of this marker observed in fibroblasts associated with normal kidneys was rather elevated and thus only resulted in a marginal statistical relationship between α-SMA expression and survival. Interestingly, comparison of the stromal expression levels of these markers in the stroma component of normal and tumor samples suggested an increase of expression in the tumor-associated stroma of all markers tested. The results for the expression of these markers in the stroma from tumor (T) vs. normal (N) based on the Mann-Whitney test were: significant for palladin (p-value = 0.016; median = 0.75 in T and 0.375 in N), marginally significant for α-SMA (p-value  = 0.078; median = 0.75 in T and 0.5 in N), significant for EDA (p-value = 0.010; median = 0.625 in T and 0.125 in N) and relatively unchanged for stroma control pan-collagen (p-value = 0.364; median = 0.50 in T and 0.50 in N). No additional correlations were observed.

**Table 1:** Types of samples represented in the tumor microarray.

Sample	n
Normal	7
Tumor	47

Patients	Percentage
Female-Male	35%-65%

Age span	Years
**Average (range)**	61.86 (39-80)

Pathology N stage	
lymph Node	N (%)
0	30 (64)
1	0 (0)
2	8 (17)
3	0
NA	9 (19)

Pathologic T stage	
Tumor	N (%)
1	14 (33)
2	8 (19)
3	15 (35)
NA	6 (14)

Pathology M Stage	
M stage	N (%)
Met	13 (30)
Non Met	28 (65)
NA	2 (5)

Pathology Stage group	
Stage	N (%)
1	13 (32)
2	6 (15)
3	5 (12)
4	14 (34)
ND	3 (7)

Characteristics of the RCC cohort used to create the tumor microarray used in this study are listed. N represents the number of cases while percentages are shown inside parentheses. Note that 62% of tumors were clear cell carcinomas.

### Stromal marker expression is 3D matrix-dependent

**Figure 2:**
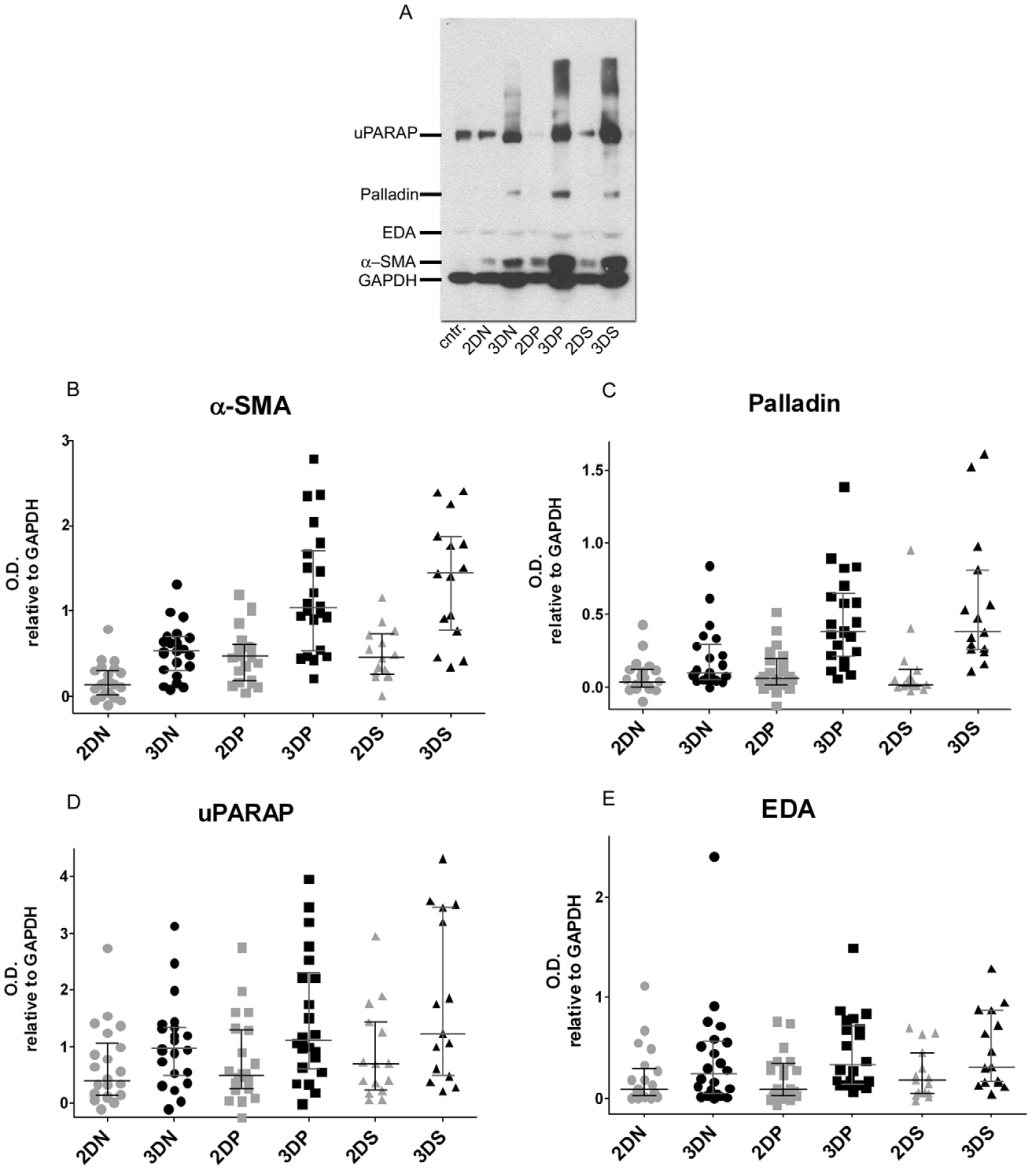
Expression levels of *in vitro* stromal markers are 3D matrix dependent. Lysates from fibroblasts cultured in 2D and 3D conditions were subjected to Western Blot analyses. **A** is a representative gel showing a fibroblastic cell line control (Wi38) and samples corresponding to fibroblasts harvested from normal kidney tissue (N), primary tumor (P) and secondary metastatic lymph node positive tumor (S) corresponding to case number #6 in Table 2. **B-E** panels show optical densities (O.D.) calculated for the assorted stromal markers relative to GAPDH. Each dot represents a single cell line. Median values as well as 25 and 75 percentiles are shown. Note the variation of the medians between 2D and 3D conditions. See Table S2 for details.

Fresh surgical samples from six selected RCC cases (Table 2 and Table S1) which rendered a total of 59 primary and immortalized fibroblastic cell lines were used for the *in vitro* portion of the study (see Materials and Methods for details regarding these cells). These fibroblasts were used to obtain 3D cultures *in vitro* using a previously published system shown to accurately mimic *in vivo* physiological behaviors of normal and tumor-associated fibroblastic cells [7], [9], [16]. In order to assess the levels of expression of the various stromal markers and to uncover the conditions needed to achieve *in vivo-*like phenotypes, Western Blot analyses were conducted using classic 2D compared to 3D cultures on the above-mentioned 59 cell lines. Quantitative analyses were conducted by measuring the marker's optical densities obtained from all cells. Figure 2A depicts an example of a typical experiment, while the graphs in Figure 2 show the compiled results where each plotted dot corresponds to a single cell line. When comparing the 3D levels of expression of all four markers to the corresponding 2D samples, we observed statistically significant fold differences in α-SMA and palladin (ranging from 2 to 20 folds) in fibroblasts associated with normal, tumor or metastatic tissues (Table S2). In contrast, uPARAP and EDA-fibronectin ratios were only significant in samples corresponding to the primary tumors but not to the normal (non-tumorigenic) or metastatic tissues (Table S2). Therefore, we concluded that fibroblastic α-SMA and palladin levels of expression *in vitro* are 3D matrix-dependent, suggesting that an *in vivo*-like relevance could be implied to the self-derived 3D microenvironmental substrates.

**Table 2:** RCC cases and paired tissues used to harvest fibroblastic cell lines.

S.N.	Tumor-type	Nomenclature	Grade	Stage	Gender/Age
1	Clear cell carcinoma	T1bN0M0	2	**I**	F/74
2	Papillary + Sarcomatoid	T3bN2M0	High	**III a**	M/49
3	Clear cell carcinoma	T3bN2M0	4	**III b**	M/49
4	Clear cell carcinoma	T3bN2M1	4	**IV a**	M/60
5	Clear cell carcinoma	T3NxM1	4	**IV b**	F/67
6	Papillary + Sarcomatoid	T3aN2M1	High	**IV c**	F/57

Six out of 22 RCCs cases were selected to harvest fibroblasts, which were used in the *in vitro* portion of the study. Sample numbers (S.N.), tumor types, nomenclatures, stages and grades are listed. Also listed are the respective gender; female (F) and male (M), along with the age of the patients at the time of surgery. Lower case indexes were assigned to the stages in order to differentiate among samples obtained from cases that were characterized at equal pathological and clinical stages.

### Assessment of levels of stromal marker expressions considering the original RCC stages

**Figure 3:**
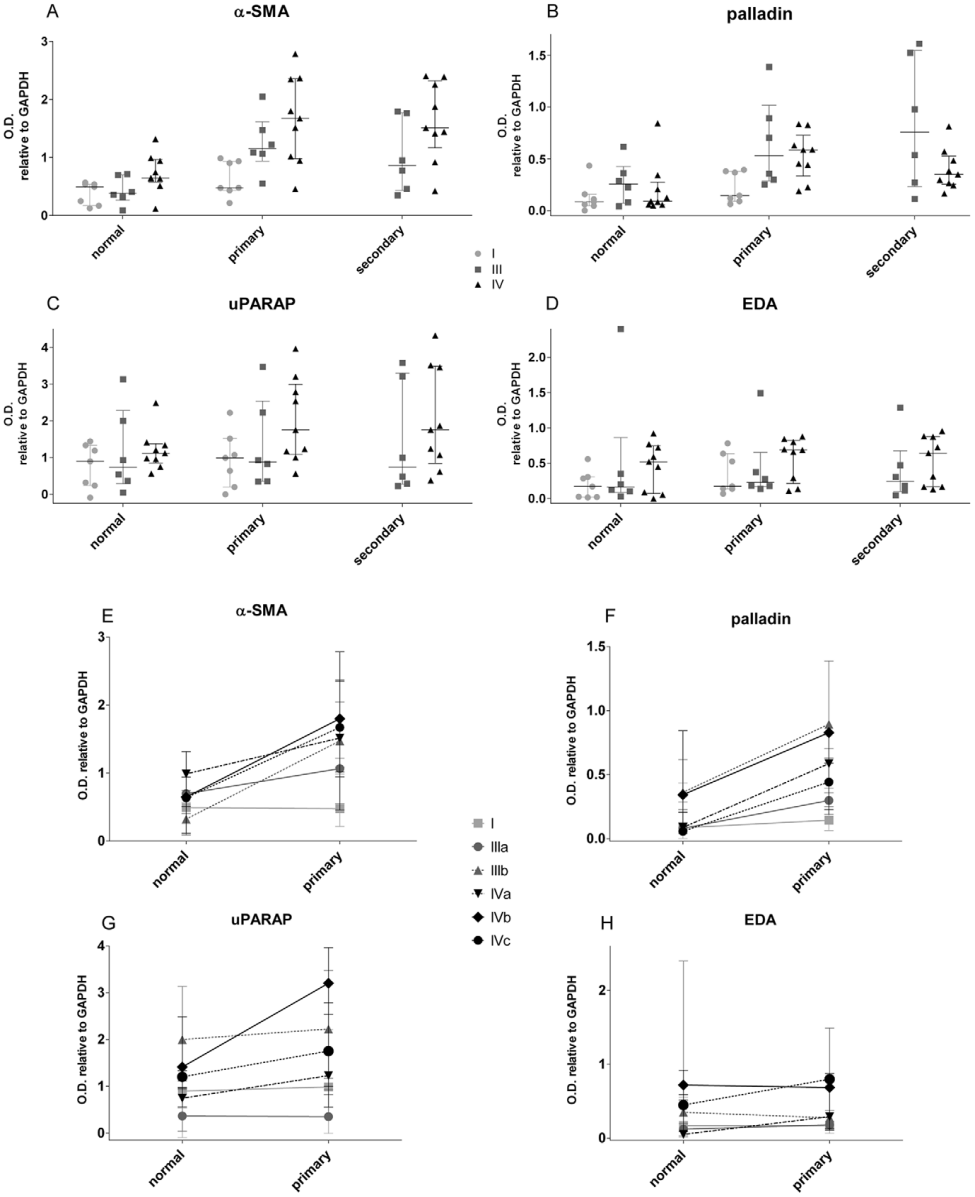
Expression levels of *in vitro* stromal markers sorted by tumor stage or by individual cases. Lysates obtained from fibroblasts cultured in 3D conditions were subjected to Western Blot analyses. Samples were sorted by tissue type (normal kidney and primary or secondary tumors) and by tumor stage. Panels **A-D** show the optical densities (O.D.) of the corresponding stromal markers relative to GAPDH. Each dot represents a single cell line. Median values as well 25 and 75 percentiles are shown. Note the variations of expression in tumor, with respect to normal, tissues as well as between early (I) and late (III and IV) stages. Sample distribution demonstrates a high level of variation. See Table S3 for details. Panels **E-H** correspond to median values and errors calculated for lysates obtained from 3D cultures of fibroblasts harvested from both normal and primary tumors sorted by the individual cases.

Since the 3D expression levels of all four markers were distributed with significantly scattered variations (Figures 2B-2E), we tested whether these could have stemmed from the original tumor-stages of the selected RCC cases. For this, the expression levels of the four markers were plotted for all *in vitro* 3D samples sorted by the corresponding original RCC stages as listed in Table 2. Figure 3 shows a clear tendency indicating that levels of expression of α-SMA and palladin (but not always uPARAP and EDA) stemming from the lower stage I case were modest when compared to the levels observed in 3D cultures from fibroblasts of higher tumor stages (Table S3). Nevertheless, the levels of α-SMA, palladin and uPARAP were distributed with larger variations of expression compared with the more even distribution of EDA expression. Cases showing higher α-SMA and/or palladin did not always correspond to the ones showing higher uPARAP or EDA (Figures 3E-3H). Moreover, within each RCC case, uPARAP and EDA expressions were not significantly changed when cultures of fibroblasts from normal kidney tissue were compared to tumor-derived ones. Interestingly, it seemed that each marker presented different patterns and that none of them truly correlated with the tumor stages (I, III or IV) or types (histological sarcomatoid (circles) or clear cell) of the original samples. Results suggest that stromal progression, although influenced by tumorigenesis, could progress at rates that are independent from their associated epithelial components as observed above in stromal palladin using TMAs.

### Sorting of stromal 3D stages using indirect immunofluorescence *in vitro*

**Figure 4:**
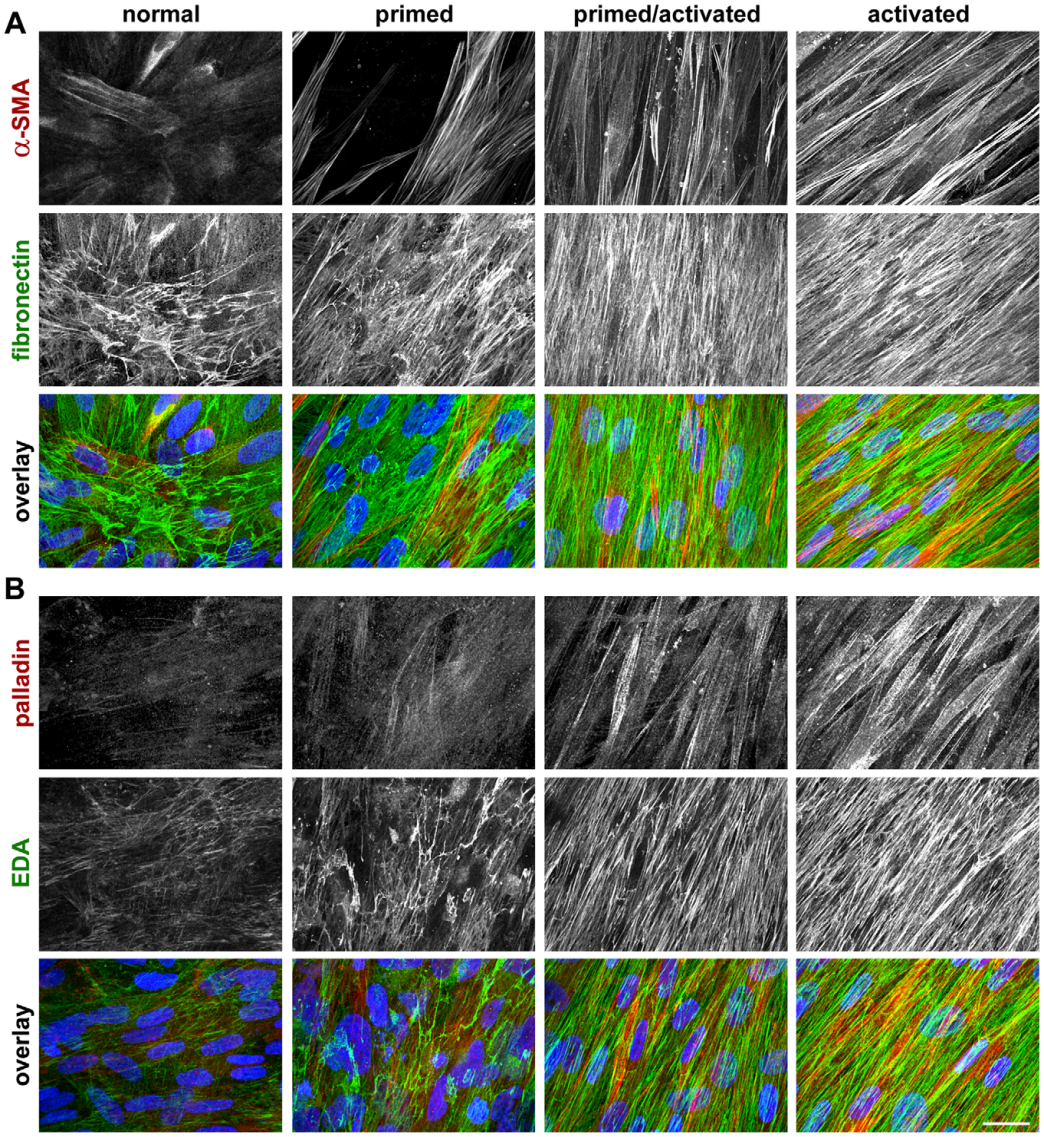
*In vivo*-like 3D kidney stroma system shows at least four progressive stromal stages. Representative reconstituted confocal images obtained from indirect immunofluorescence of selected un-extracted 3D cultures. **A** depicts samples labeled with antibodies against α-SMA and fibronectin while **B** depicts palladin and EDA-fibronectin. Note that the homogeneity and expression along stress fibers of both α-SMA and palladin is incremental for normal (i.e., cells harvested from the non-tumorigenic tissue of case #5 in Table 2), primed (cells isolated from the primary tumor sample of case case #1 in Table 2), primed/activated (cells used were harvested from the primary tumor sample of case case #2 in Table 2) and activated (cells were harvested from the primary tumor sample of case #5 in Table 2) stages. In addition, parallel patterned organization of matrices labeled with fibronectin (**A**) and EDA (**B**) are also incremental while EDA-fibronectin also shows incremental increases in expression levels. Overlaid images, in A and B respectively, contain α-SMA or palladin in red, fibronectin or EDA in green and nuclei in blue. Bar represents 25 µm.

In order to test whether measured expression levels of stromal markers in 3D cultures correlate to their subcellular localization and architecture, we conducted indirect immunofluorescence as in a previous publication [8]. We assessed the topographical features of cellular fibronectin fibers or of its splice variant EDA looking at activated or tumor associated parallel-organized signature fiber patterns, as opposed to the mesh-like and disorganized patterns formed by these fibers in the cultures from fibrobalsts derived from normal (i.e., non-activated) stage [7], [8], [9], [17]. Hence, localized stress fiber and homogenous patterns of α-SMA and palladin characterize the activated (or tumor-associated) stromal stage, while heterogeneous and cytosolic patterns are consistent with the non-activated stromal stages [12], [18], [19], [20]. Figure 4 shows representative images of four identified stromal stages regarded as i) ‘normal’ or non-activated, ii) ‘primed,’ iii) ‘primed/activated’ or intermediate and iv) ‘activated’ or tumor-associated. Analyses revealed that the pattern ascribed to activated stromal stages did not always correspond to the advanced tumor stages, suggesting that the progression of the stroma may be a somewhat independent feature from the well established designated tumor stage similar to the case of stromal palladin *in vivo* using the TMAs. Nevertheless, the clear correlation between the immunofluorescent analyses of 3D stromal cultures to their corresponding Western Blots with regard to α-SMA and palladin levels supported the conclusion that stages assessed by indirect immunofluorescence may be predicted by the corresponding Western Blot results.

### *In vitro* stroma stages, assessed by 3D cultures, correlate to *in vivo* equivalents

**Figure 5:**
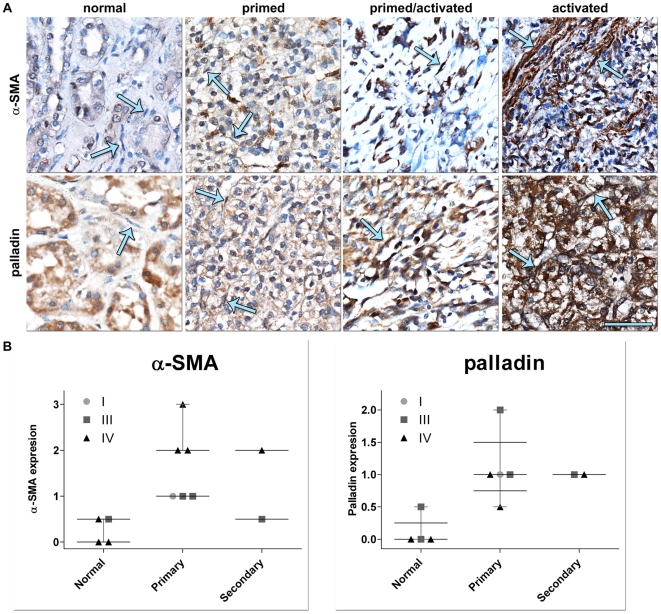
*In vivo* kidney stroma is sorted into progressive stages correlating the ones observed *in vitro*. Representative α-SMA and palladin immunohistochemistry (**A**) labeled images matching samples shown in Figure 4 corresponding to Table 2 cases: #5 for both normal or non-tumorigenic (normal stroma) and tumor (activated stroma), #1 (primed stroma) and #2 (primed/activated or intermediate stroma). Arrows in **A** are pointing to stromal cells. Barr represents 50 µm. Note that, although palladin is localized to the epithelial components of the tissue samples, the stromal marker expression and patterning of both α-SMA and palladin are incremented according to the stage of the stroma. Graphs in **B** represent the *in vivo* stromal levels of expression of α-SMA and palladin sorted by tumor stage and by tissue type (medians and distributions are shown). See Table S4 for details.

In order to validate the *in vitro* sorting of 3D cultures into progressive stromal stages, paraffin embedded tissue sections corresponding to the cases used in the *in vitro* analyses were immunostained for α-SMA, palladin, uPARAP, and EDA-fibronectin. The expression levels of these markers in the tumor-associated stroma were assessed using a blinded scoring approach (as done for the TMAs). Representative results are shown in Figure 5A, while observed expression level values are plotted in Figure 5B and listed in Table S4. Stroma of normal kidney tissue presented only low expression or no expression levels of these markers. Immunohistochemistry analyses utilizing uPARAP and EDA were not specific enough and therefore were inconclusive for these analyses. Notably, results observed for α-SMA and palladin *in vitro* showed expression levels and localization patterns that corresponded to their *in vivo* counterparts (compare results in Figure 5 to results in Figures 3 and 4).

## Discussion

Several studies, including some looking at renal cancers, point to stromal components as key players in inducing tumorigenesis and in the acquisition of invasive behaviors [5], [21], [22], [23], [24], [25], [26], [27]. Hence, investigators have suggested that analysis of the tumor stroma may render meaningful prognostic data [28], [29], [30], [31], [32], [33]. In this context, targeting tumor-associated stroma may hamper its induced permissiveness towards epithelial cell transformation and/or invasion and, consequently, increase the efficacy of chemo- and radiotherapies [34]. It is well established that tumor activated fibroblast express myofibroblastic markers, such as α-SMA [35], palladin [20] uPARAP/Endo180 [15] and EDA-fibronectin [3]. As the role of these stromal markers in RCC progression is currently unknown, we investigated the association between the expression of these markers and RCC prognosis.

In a recent collaborative work, we demonstrated that increased expression of stromal palladin can be found in fibroblasts associated with pancreatic ductal adenocarcinoma and other neoplasias such as lung, skin, breast and kidney [20]. Therefore, and as previously suggested [36], one could speculate that palladin expression may alter some key stromal properties that induce or increase tumorigenesis. To this end after analyzing our TMA data, we concluded that stromal expression of palladin correlated with the survival of RCC patients; higher levels of stromal predicted lower overall survival times. Hence, palladin may constitute an independent marker for prognosis. Strikingly, the predictive value of this stromal marker was highly significant in patients who had not developed metastasis suggesting that it could constitute a high risk marker in this patient population. Another stromal marker whose expression correlated with stromal activation, α-SMA, showed a rather high background staining in the stroma associated to normal kidney tissue resulting in less impressive differences with the stroma associated with tumors. In addition, the reduced number of samples for which survival data are available decreased the statistical significance between the mean staining score between these two populations. In this context, palladin seemed to be a stronger predictor of survival than α-SMA since the correlation showed by comparing palladin staining in stroma associated with normal or tumoral tissue remained statistically significant in spite of the few samples whose survival data were not available.

The expression of markers suggestive of stromal activation could serve as indicators of tumor-stromal interactions [22], a largely understudied aspect of RCC. Palladin plays important roles in actin organization, sarcomere integrity, and cytoskeleton architecture. Palladin has also been associated with stimulation of cell motility and increased matrix stiffness, both characteristics attributed to tumor-associated activated stroma [37]. To address changes associated with the RCC stroma, we developed a novel renal fibroblast derived 3D culture system that, similar to other systems [7], [9], [16], [22], effectively mimics many aspects of the *in vivo* stromal microenvironments. We used fibroblasts harvested from five late RCC stage cases with matching non-activated (i.e., normal), primary and metastatic tumors and one localized early stage case as a control. We observed that self-derived 3D cultures recapitulate the original (i.e., *in vivo*) expression levels of the four selected markers. Moreover, 3D matrix-induced α-SMA and palladin *in vitro* levels greatly correlated with the stromal activation of fibroblasts (myofibroblasts). On the other hand, uPARAP and EDA-fibronectin expressions appeared to be regulated independently of 3D ECMs suggesting that perhaps their expression depends on additional parameters such as tissue type or cellular location. Previous reports from our laboratory indicated that, in order to achieve *in vivo*-like stromal expression patterns, culturing cells onto fibroblast-derived matrices or as un-extracted 3D cultures, is often necessary [7], [9]. By the use of these 3D cultures, we demonstrated that levels of stromal markers α-SMA, palladin and, to some extent, uPARAP are consistently and significantly increased compared to those of all harvested from normal kidney tissues, reflecting their usefulness as surrogates of *in vivo* specimens. On the other hand, the fibronectin spliced form EDA did not show significant variations.

In spite of the clear differences in the levels of expression of α-SMA and palladin observed when comparing 3D cultures produced by tumor-derived vs. normal kidney-derived fibroblasts, relatively high distributions among samples were also observed. To this end, it has been suggested that myofibroblastic activation occurs in progressive phases [18] and that perhaps tumor-associated stroma can also be sorted into various progressive stages [21], [35]. In an effort to determine whether incorporating stromal features into an enhanced staging scheme could be used for RCCs, we investigated whether stroma progression accompanies or rather develops at an independent pace from tumor staging. We questioned if our 3D culturing system effectively mimics the original *in vivo* stromal features. Individual analysis of RCC cases indicated that levels of stromal markers do not necessarily always correlate with the original stages of the tumors. Nevertheless, RCC cases showing the greatest expression levels of both α-SMA and palladin indeed correlated with the most activated 3D cultures sorted using our published methods [7], [8], [9]. Interestingly, the same cases sorted as ‘activated’ presented *in vivo* characteristics of activated stroma such as increased levels of stromal markers palladin and α-SMA indicating that our 3D system indeed mimics *in vivo* stromal characteristics.

Our results suggest that the rate of stroma transformation does not correlate with the tumor stage assigned by the evaluation of the tumor cells proposing the possibility that the assessment of stroma progression may complement tumor stage as a clinical prognostic variable in RCC. Moreover, we believe that we have identified stromal palladin as an early risk factor predictor for RCCs.

## Materials and Methods

### Ethics Statement

All human tissues used in this study were acquired after approval of the Fox Chase Cancer Center's Institutional Review Board. All samples were decoded to avoid the possibility of patient identification. It is important to note that a written informed consent form, where patients agree to have samples taken for research purposes, was obtained in all the cases.

### Reagents

Dulbecco's modified Eagle's medium (DMEM) was obtained from Mediatech Inc. (Manasas, VA) and fetal bovine serum (FBS) from Hyclone (South Logan, UT). Mouse anti-α-SMA and rabbit anti-fibronectin were from Sigma-Aldrich (St. Louis, MO). Mouse anti-EDA fibronectin was obtained from Abcam (Cambridge, MA) and rabbit anti-palladin (for immunofluorescence and immunohistochemistry) from Proteintech group (Chicago, IL). Monoclonal mouse anti-palladin (used in Western Blots) was a gift from Dr. C. Otey from the University of North Carolina [12], [38], and mouse anti-uPARAP was a gift from Drs. T. Bugge (NIDCR/NIH, Bethesda, Maryland) and L. Engelholm (Copenhagen Biocenter, Denmark) [15]. Rabbit anti-Vimentin was from Biovision (Mountain View, CA), mouse anti-pan-keratin from Abcam (Cambridge, MA) and mouse anti-GAPDH from Millipore. Goat anti-mouse IRDye 700 and anti-rabbit IRDye 800 were from LI-COR Biosciences (Lincoln, NE). Anti-mouse Rhodamine red and anti-rabbit Cy5, donkey F(ab')2 fragments were from Jackson Laboratories (West Grove, PA). Plasmids 12245 (pLOX-TERT-iresTK) and 12240 (pLOX-CWBmi1) were from Addgene (Cambridge, MA, USA). FuGENE 6 Transfection Reagents were from Roche Diagnostic (Indianapolis, IN, USA).

#### RCC patient information for tumor microarray analysis

53 RCCs samples were collected by the Tumor Bank and Bio-specimen Repository Facilities at Fox Chase Cancer Center following IRB approval. Among the 53 RCC cases, 47 constituted malignant tumor samples while 7 represented normal tissues used as controls. Amid the 47 tumor cases, 62% of the cases were clear cell carcinomas. A detailed description of the cohort is presented in Table 1.

### Immunohistochemistry

Immunohistochemistry of paraffin-embedded samples was performed using rabbit anti-palladin (1∶100) or mouse anti-α-SMA (1∶100) as primary antibodies. An avidin biotin-peroxide kit (Vectastain Elite; Vector Laboratories, Burlingame, CA), together with chromogen 3′,3′-diaminobenzidine, was used following manufacturer's instructions. Negative controls consisted of treated samples incubated using iso-matched non-specific primary antibodies and normal rabbit or mouse pre-immune sera. All sections were counterstained with hematoxylin and mounted for inspection.

The score for the intensity of staining in the immunohistochemistry was performed by a ‘blinded’ pathologist using semiquantitative measurements. The assigned intensities consisted of four categories; 0, 0.5, 1, and 2. Absence of staining was assigned with the lowest value (0) and the strongest set of intensities was assigned the highest (2). The correlation between immunohistological staining and clinical parameters such as presence of local or distant metastases, and survival was analyzed by a biostatistician (see below).

### Isolation of primary fibroblasts from surgical samples

Fresh surgical tissue samples from partial nephrectomies conducted at the Fox Chase Cancer Center were delivered with the assistance of the Protocols Laboratory and Biosample Repository Facility following protocols approved by the Institutional Review Board. Twenty-two primary RCCs, 18 normal (non-tumorigenic) kidney samples and 5 secondary tumors (positive lymph nodes) were obtained (Table S1). Tissue samples were rinsed in cold PBS containing 100 U/ml penicillin and 100 µg/ml streptomycin. Samples were minced and subjected to overnight digestive dissociation using 0.2% collagenase at 37°C. Note that samples that were big enough were divided in two separate harvesting samples at this point. The resultant mixture was centrifuged at 200 g for 4 min. Supernatants were passed through 100 µm cell strainer (BD Bioscience) before plating. The fibroblasts-enriched fraction was cultured for up to 6 passages in DMEM containing 15% FBS, 100 U/ml penicillin, 100 µg/ml streptomycin and 2 mM L-glutamine at 37°C using a humidified atmosphere and 5% CO_2_. The resultant lines were regarded as primary cell lines which served as parental for the immortalization ones (see below). Homogeneity was confirmed by direct microscopic observations, and cells were designated as fibroblastic after confirmation of mesenchymal marker vimentin expression, as well as absence of epithelial marker keratin expression. WI38 and MCF7 cells were used as fibroblastic positive and negative controls, respectively (Table S1).

### Selection of stromal fibroblasts harvested from normal and matched RCC tissues

Seventy-two primary fibroblastic human kidney cell lines were obtained from the 22 RCC cases listed in Table S1. The fibroblastic nature and the level of homogeneity of all cells were assessed as stated above. Vimentin-positive keratin-negative lines were kept as primary lines and also served as parental lines for immortalization by transfection using lipofectamine (Roche, Indianapolis, IN, USA) with plasmids 12245 (pLOX-TERT-iresTK) and 12240 (pLOX-CWBmi1) and incubated in OPTI-MEM (GIBCO) reduced serum according to the manufacturers' instructions [39]. On average, two immortalized cell lines were obtained for each selected parental primary one. Successful transfections were confirmed by Western Blotting assessing high Bmi1 [40] and low p16 expressions. Six representative cases were selected (Table 2 and Table S1). The selected cells spanned a total of 59 lines from which 17 were primary (parental) cells and 42 corresponded to immortalized sets. Five of the 6 selected included fibroblasts derived from paired samples of normal (non-tumorigenic) kidney tissue, as well as matched adjacent primary and secondary (e.g., positive lymph nodes or adrenal gland) tumors. The sixth case, which included cells harvested only from normal kidney and adjacent primary tumor, corresponded to a low grade, low stage (T1b, grade 2) clear cell RCC used as a control. Four of the six selected cases were histologically assessed as clear cell carcinomas, while two were identified as papillary with sarcomatoid in RCC features. In all, the selected cases contained two stage III and three stage IV representatives while one was deemed low nuclear grade and (grade 2) at stage I (Table 2). Experiments were performed twice using both immortalized and primary lines.

### Fibroblast-derived ECM based 3D cultures

Protocol was as previously described [8], [41]. Briefly, 250,000 cells/ml were plated on gelatin-coated dishes or cover-slips and maintained in a state of confluence for 6-8 days. The gelatin served to stabilize the fibronectin matrices derived by cells cultured onto this pre-coated surface. This way the cell-derived matrices build in a multilayer fashion resulting in stable structures which could withstand the intrinsic forces applied by the cells during 3D matrix production in the layers. Cells were supplemented every 48 hours with 50 µg/ml L-ascorbic acid (this also helps to stabilize matrix production so cells can continue building up). The resultant “unextracted ECM based 3D cultures” were either lysed for Western Blot analyses or processed for indirect immunofluorescence. Controls consisted of matched sample cultures grown overnight under classic 2D conditions.

### Western Blot

Cells were lysed and samples were resolved by SDS-PAGE and transferred as previously described [7], [8]. Blots were incubated with a combination of primary antibodies against α-SMA (1∶1000), palladin (1∶1000), EDA-fibronectin (1∶1000), uPARAP (1∶1000) and GAPDH (1∶2000) or Vimentin (1∶1000) and pan-Keratin (1∶1000). IRDye 700 goat anti-mouse and IRDye 800 goat anti-rabbit (1∶10,000, LI-COR) were used as secondary antibodies and blots were scanned using the Odyssey Infrared Imaging System (LI-COR) following the Odyssey User Guide's (version 2.1) instructions for membranes. Optical densities were obtained using the Odyssey 2.1.12 software with median background subtraction and 3 points selected border width. Ratios showing relative levels of expression with regards to GAPDH were calculated and plotted using Prism 5 software (GraphPad Software, San Diego, CA).

### Indirect immunofluorescence

Samples were labeled according to standard para-formaldehyde/triton procedures [7], [8], [16]. Briefly, samples were incubated at room temperature with either rabbit anti-human fibronectin (1∶100) and mouse anti-α-SMA (1∶100) or mouse anti-EDA-fibronectin (1∶100) and rabbit anti-palladin (1∶100). Samples were rinsed and incubated with secondary anti-rabbit Cy5 and anti-mouse Rhodamine Red conjugated donkey F(ab')2 fragments (1∶100) together with SYBR Green nuclear stain (1∶50,000, Invitrogen) for 30 minutes at room temperature. Samples were rinsed and then mounted using Invitrogen's Prolong gold anti-fading reagent (Carlsbad, CA, USA).

### Confocal Image Acquisition and Reconstitution

Samples were scanned using an Ultraview spinning-disc confocal head (Perkin-Elmer Life Sciences, Boston, MA) mounted on a Nikon TE-2000U microscope (Optical Apparatus Co., Ardmore, PA). Images, corresponding to simultaneous 488 nm, 568 nm, and 647 nm wavelengths, were captured using sequential Z-slices corresponding to 0.5 µm. Reconstituted projections were obtained by applying a maximum reconstruction function onto the acquired Z-planes using MetaMorph Offline 7.0r1 imaging analysis software (Molecular Devices, Downingtown, PA).

### Statistical Analyses

Statistical analyses for Western blot and for normal vs. tumor immunohistochemistry were performed using the non-parametric Mann-Whitney test calculated by the Instat statistical software (GraphPad Software, San Diego, CA). Regarding the tumor micro array, in order to identify clinical variables related to patient survival, we performed univariate and multivariable analyses by constructing decision trees using the Classification and Regression Trees (CART) methodology. The following clinical variables were considered as predictors of survival time – levels of the markers α-sma, palladin, fibronectin EDA and collagen I, as well as T stage, N stage, M stage and pathological stage. We utilized the unified CART framework that embeds recursive binary partitioning into the theory of permutation tests [42]. Significance testing procedures were applied to determine whether no significant association between any of the clinical variables and the response could be stated or whether the recursion would need to stop. We utilized the open-source R package PARTY (www.r-project.org) in our computations [43]. Due to the nature of our analyses, no correction for multiple testing was employed, and a Type I error of 5% was used to test each hypothesis.

## Supporting Information

Table S1 **RCC cases and their paired/harvested cell lines.** List spanning the 22 RCCs cases, tumor types, nomenclatures and collaborative (pathological and clinical) stages and grades used in this study. Also listed are the types of tissues from where fibroblasts were isolated (e.g., normal kidney as well as primary and secondary (lymph node or adrenal gland) tumors). K, stands for keratin while V stands for vimentin. Harvested cells were sorted by their morphological features as spread, intermediate or spindled. ND stands for not determined. * indicate samples selected for the rest of the study while **a**, **b** and **c** in the stage column are used to distinguish between the two stage III and three stage IV samples among the six selected.
(DOC)

Table S2 **Medians and statistical P values for measured Optical Densities.** Median calculated optical densities normalized to GAPDH values are shown in A, while B-E correspond to the P values obtained using Mann-Whitney test. Relative P value significances were designated as extremely***, very**, or significant*. The tissue sources from where fibroblasts were harvested are marked as **N** for normal kidney, **P** for primary RCC and **S** for secondary (metastatic) RCC. 2D and 3D correspond to two-dimensional and three-dimensional cultures, respectively.
(DOC)

Table S3 **Calculated medians, fold differences and statistical P values for 3D cultures sorted using the original RCC's stages.** Median calculated optical densities normalized to GAPDH values are shown in A, while B-E correspond to the indicated median fold differences and corresponding P values obtained using the Mann-Whitney test. Relative P value significances were designated as extremely***, very**, or significant*. Roman numbers (I, III and IV) correspond to the original collaborative tumor stages from where fibroblasts were harvested. The tissue sources rendering the fibroblasts used in the study are marked as N for normal kidney, P for primary RCC and S for secondary (metastatic) RCC.
(DOC)

Table S4 **Expression levels of** ***in vivo*** **stroma markers α-SMA and palladin analyzed by immunohistochemistry.** Table listing collaborative stages where * corresponds to samples used for *in vitro* analyses while **a**, **b** and **c** serve to differentiate among the cases as in Table 1. Types of tissues used are depicted as normal, as well as primary or secondary for tumors. Blinded assessment of immunohistochemistry expression levels using -, -/+, +, ++ and +++ as scoring method. The blinded individual explained the observed stroma (as opposed to epithelial) positive staining of all samples under “Description of stromal expression.”
(DOC)
