# Supplementary material for: Elevated Expression of Stromal Palladin Predicts Poor Clinical Outcome in Renal Cell Carcinoma
Source: PLoS One. 2011 Jun 28;6(6):e21494. doi: 10.1371/journal.pone.0021494 (PMC3125241; doi:10.1371/journal.pone.0021494)
Supplement: Table S1 — RCC cases and their paired/harvested cell lines. List spanning the 22 RCCs cases, tumor types, nomenclatures and collaborative (pathological and clinical) stages and grades used in this study. Also listed are the types of tissues from where fibroblasts were isolated (e.g., normal kidney as well as primary and secondary (lymph node or adrenal gland) tumors). K, stands for keratin while V stands for vimentin. Harvested cells were sorted by their morphological features as spread, intermediate or spindled. ND stands for not determined. * indicate samples selected for the rest of the study while a, b and c in the stage column are used to distinguish between the two stage III and three stage IV samples among the six selected. (DOC) [file pone.0021494.s001.doc]

**Table S1: RCC cases and their paired/harvested cell lines.**

| **S.N.** | **Tumor-type** | **Nomenclature** | **Stage** | **Grade** | **Tissue** | **K** | **V** | **Morphology** |
| --- | --- | --- | --- | --- | --- | --- | --- | --- |
| 1 | Clear cell | T1bN0M1 | IV | 2 | Primary Tumor | + | + | N/A |
| 2 | Clear cell | T3bN0M1 | IV | 4 | Primary Tumor | + | + | N/A |
| 3 | Clear cell | T1aN0M0 | I | 3 | Primary Tumor | + | + | N/A |
| 4 | Clear cell | T1bN0M0 | I | 2 | Primary Tumor | + | + | N/A |
| 5 | Clear cell | T3aN0M0 | I | 3 | Primary Tumor | - | + | Intermediate |
| 6 | Sarcomatoid in RCC | T3bN0M0 | III | high | Primary Tumor | - | + | Intermediate |
| 7 | Clear cell | T1bN0M0 | I | 3 | Normal Kidney | + | + | Spread |
|  |  |  |  |  | Primary Tumor | - | + | Intermediate |
| 8 | Papillary | T1bN0M0 | I | high | Normal Kidney | + | - | Spread |
|  |  |  |  |  | Primary Tumor | - | + | Intermediate |
| 9 | Clear cell | T3bN0M0 | III | 3 | Normal Kidney | + | + | Spread |
|  |  |  |  |  | Primary Tumor | - | + | Intermediate |
| 10 | Clear cell | T3cN0M0 | III | 2 | Normal Kidney | + | + | Spread |
|  |  |  |  |  | Primary Tumor | - | + | Intermediate |
| 11 | Clear cell | T2N0M0 | II | 2 | Normal Kidney | + | + | ND |
|  |  |  |  |  | Primary Tumor | + | + | ND |
| 12 | Papillary | T2N0M0 | II | high | Normal Kidney | - | + | Spread |
|  |  |  |  |  | Primary Tumor | + | + | Epithelial |
| 13 | Clear cell | T3bN0M0 | III | 3 | Normal Kidney | + | - | ND |
|  |  |  |  |  | Primary Tumor | - | + | Intermediate |
| 14 | Chromophobe | T3aN0M0 | III | ND | Normal Kidney | + | - | ND |
|  |  |  |  |  | Primary Tumor | - | + | Intermediate |
| 15 | Papillary | T2N0M0 | II | high | Normal Kidney | + | + | ND |
|  |  |  |  |  | Primary Tumor | - | + | Intermediate |
| 16 | Clear Cell | T1aN0M0 | I | 2 | Normal Kidney | + | + | ND |
|  |  |  |  |  | Primary Tumor | + | + | ND |
| 17 * | Clear cell | T1bN0M0 | I | 2 | Normal Kidney | - | + | Spread |
|  |  |  |  |  | Primary Tumor | - | + | Intermediate |
| 18 * | Clear cell | T3bN2M1 | IV **a** | 4 | Normal Kidney | - | + | Spread |
|  |  |  |  |  | Primary Tumor | - | + | Spindle |
|  |  |  |  |  | Secondary Tumor | - | + | Spindle |
| 19 * | Sarcomatoid in RCC + Papillary | T3bN2M0 | III **a** | high | Normal Kidney | - | + | Spread |
|  |  |  |  |  | Primary Tumor | - | + | Intermediate |
|  |  |  |  |  | Secondary Tumor | - | + | Spindle |
| 20 * | Clear cell | T3bN2M0 | III **b** | 4 | Normal Kidney | - | + | Intermediate |
|  |  |  |  |  | Primary Tumor | - | + | Spindle |
|  |  |  |  |  | Secondary Tumor | - | + | Intermediate |
| 21 * | Clear cell | T3NxM1 | IV **b** | 4 | Normal Kidney | - | + | Spread |
|  |  |  |  |  | Primary Tumor | - | + | Spindle |
|  |  |  |  |  | Secondary Tumor | - | + | Spindle |
| 22 * | Sarcomatoid in RCC + Papillary | T3aN2M1 | IV **c** | high | Normal Kidney | - | + | Spread |
|  |  |  |  |  | Primary Tumor | - | + | Spindle |
|  |  |  |  |  | Secondary Tumor | - | + | Intermediate |

List spanning the 22 RCCs cases, tumor types, nomenclatures and collaborative (pathological and clinical) stages and grades used in this study. Also listed are the types of tissues from where fibroblasts were isolated (e.g., normal kidney as well as primary and secondary (lymph node or adrenal gland) tumors). K, stands for keratin while V stands for vimentin. Harvested cells were sorted by their morphological features as spread, intermediate or spindled. ND stands for not determined. *indicate samples selected for the rest of the study while **a**, **b** and **c** in the stage column are used to distinguish between the two stage III and three stage IV samples among the six selected.
