# Supplementary material for: Elevated Expression of Stromal Palladin Predicts Poor Clinical Outcome in Renal Cell Carcinoma
Source: PLoS One. 2011 Jun 28;6(6):e21494. doi: 10.1371/journal.pone.0021494 (PMC3125241; doi:10.1371/journal.pone.0021494)
Supplement: Table S2 — Medians and statistical P values for measured Optical Densities. Median calculated optical densities normalized to GAPDH values are shown in A, while B-E correspond to the P values obtained using Mann-Whitney test. Relative P value significances were designated as extremely***, very**, or significant*. The tissue sources from where fibroblasts were harvested are marked as N for normal kidney, P for primary RCC and S for secondary (metastatic) RCC. 2D and 3D correspond to two-dimensional and three-dimensional cultures, respectively. (DOC) [file pone.0021494.s002.doc]

**Table S2**: Medians and statistical P values for measured Optical Densities.

| **A. Median** | | **2DN** | | **3DN** | | **2DP** | | **3DP** | **2DS** | | **3DS** | |
| --- | --- | --- | --- | --- | --- | --- | --- | --- | --- | --- | --- | --- |
| **α-SMA** | | 0.148 | | 0.535 | | 0.472 | | 1.041 | 0.456 | | 1.440 | |
| **palladin** | | 0.040 | | 0.100 | | 0.069 | | 0.387 | 0.020 | | 0.383 | |
| **uPARAP** | | 0.395 | | 0.965 | | 0.492 | | 1.119 | 0.697 | | 1.237 | |
| **EDA** | | 0.085 | | 0.240 | | 0.094 | | 0.462 | 0.246 | | 0.482 | |
| **B. α-SMA**  **P values** | **3DN** | | **2DP** | | **3DP** | | **2DS** | | | **3DS** | |  |
| **2DN** | 0.0001*** | | 0.0003*** | | 0.0001*** | | 0.0015** | | | 0.0001*** | |  |
| **3DN** | - | | 0.6 | | 0.0008*** | | 0.6 | | | 0.0005*** | |  |
| **2DP** | - | | - | | 0.0003*** | | 1.0 | | | 0.0004*** | |  |
| **3DP** | - | | - | | - | | 0.0009*** | | | 0.6 | |  |
| **2DS** | - | | - | | - | | - | | | 0.0002*** | |  |
| **C. palladin**  **P values** | **3DN** | | **2DP** | | **3DP** | | **2DS** | | | **3DS** | |  |
| **2DN** | 0.01* | | 0.3 | | 0.0001*** | | 1.0 | | | 0.0001*** | |  |
| **3DN** | - | | 0.1 | | 0.0006*** | | 0.02* | | | 0.0007** | |  |
| **2DP** | - | | - | | 0.0001*** | | 0.3 | | | 0.0001*** | |  |
| **3DP** | - | | - | | - | | 0.0001*** | | | 0.7 | |  |
| **2DS** | - | | - | | - | | - | | | 0.0001*** | |  |
| **D. uPARAP**  **P values** | **3DN** | | **2DP** | | **3DP** | | **2DS** | | | **3DS** | |  |
| **2DN** | 0.06 | | 0.7 | | 0.005** | | 0.3 | | | 0.006** | |  |
| **3DN** | - | | 0.1 | | 0.3 | | 0.4 | | | 0.2 | |  |
| **2DP** | - | | - | | 0.01* | | 0.7 | | | 0.02* | |  |
| **3DP** | - | | - | | - | | 0.1 | | | 0.6 | |  |
| **2DS** | - | | - | | - | | - | | | 0.07 | |  |
| **E. EDA**  **P values** | **3DN** | | **2DP** | | **3DP** | | **2DS** | | | **3DS** | |  |
| **2DN** | 0.1 | | 0.8 | | 0.002** | | 0.3 | | | 0.006** | |  |
| **3DN** | - | | 0.2 | | 0.1 | | 0.6 | | | 0.2 | |  |
| **2DP** | - | | - | | 0.005** | | 0.7 | | | 0.01* | |  |
| **3DP** | - | | - | | - | | 0.05* | | | 0.8 | |  |
| **2DS** | - | | - | | - | | - | | | 0.06 | |  |

Median calculated optical densities normalized to GAPDH values are shown in A, while B-E correspond to the P values obtained using Mann-Whitney test. Relative P value significances were designated as extremely***, very**, or significant*. The tissue sources from where fibroblasts were harvested are marked as **N** for normal kidney, **P** for primary RCC and **S** for secondary (metastatic) RCC. 2D and 3D correspond to two-dimensional and three-dimensional cultures, respectively.
