# Supplementary material for: Elevated Expression of Stromal Palladin Predicts Poor Clinical Outcome in Renal Cell Carcinoma
Source: PLoS One. 2011 Jun 28;6(6):e21494. doi: 10.1371/journal.pone.0021494 (PMC3125241; doi:10.1371/journal.pone.0021494)
Supplement: Table S3 — Calculated medians, fold differences and statistical P values for 3D cultures sorted using the original RCC's stages. Median calculated optical densities normalized to GAPDH values are shown in A, while B-E correspond to the indicated median fold differences and corresponding P values obtained using the Mann-Whitney test. Relative P value significances were designated as extremely***, very**, or significant*. Roman numbers (I, III and IV) correspond to the original collaborative tumor stages from where fibroblasts were harvested. The tissue sources rendering the fibroblasts used in the study are marked as N for normal kidney, P for primary RCC and S for secondary (metastatic) RCC. (DOC) [file pone.0021494.s003.doc]

**Table S3**: Calculated medians, fold differences and statistical P values for 3D cultures sorted using the original RCC’s stages.

| **A. Median** | **N I** | | **P I** | | **N III** | | **P III** | | **S III** | | **N IV** | | **P IV** | | **S IV** |
| --- | --- | --- | --- | --- | --- | --- | --- | --- | --- | --- | --- | --- | --- | --- | --- |
| **α-SMA** | 0.492 | | 0.476 | | 0.382 | | 1.153 | | 0.863 | | 0.648 | | 1.672 | | 1.509 |
| **palladin** | 0.085 | | 0.145 | | 0.256 | | 0.531 | | 0.758 | | 0.090 | | 0.586 | | 0.349 |
| **uPARAP** | 0.894 | | 0.988 | | 0.734 | | 0.877 | | 0.737 | | 1.114 | | 1.751 | | 1.756 |
| **EDA** | 0.169 | | 0.171 | | 0.160 | | 0.227 | | 0.242 | | 0.518 | | 0.686 | | 0.642 |
| B. α-SMA **median fold**  P value | | **P I** | | **N III** | | **P III** | **S III** | **N IV** | | **P IV** | | **S IV** | |  | |
| N I | | **1.0** | | **0.8** | | **-** | **-** | **1.3** | | **-** | | **-** | |  | |
|  |  | 0.4 | | 0.8 | | - | - | 0.02* | | - | | - | |  | |
| P I | | **-** | | **-** | | **2.4** | **1.8** | **-** | | **3.5** | | **3.2** | |  | |
|  |  | - | | - | | 0.008** | 0.4 | **-** | | 0.005** | | 0.01* | |  | |
| N III | | **-** | | **-** | | **3.0** | **2.3** | **1.7** | | **-** | | **-** | |  | |
|  |  | - | | - | | 0.009** | 0.06 | 0.1 | | **-** | | **-** | |  | |
| P III | | **-** | | **-** | | **-** | **0.7** | **-** | | **1.5** | | **1.3** | |  | |
|  |  | - | | - | | - | 0.4 | **-** | | 0.4 | | 0.3 | |  | |
| S III | | **-** | | **-** | | **-** | **-** | **-** | | **2.0** | | **1.7** | |  | |
|  |  | - | | - | | - | - | - | | 0.1 | | 0.1 | |  | |
| N IV | | **-** | | **-** | | **-** | **-** | **-** | | **2.6** | | **2.3** | |  | |
|  |  | - | | - | | - | - | - | | 0.008** | | 0.008** | |  | |
| P IV | | **-** | | **-** | | **-** | **-** | **-** | | **-** | | **0.9** | |  | |
|  |  | - | | - | | - | - | - | | - | | 0.9 | |  | |
| C. palladin **median fold**  P value | | **P I** | | **N III** | | **P III** | **S III** | **N IV** | | **P IV** | | **S IV** | |  | |
| N I | | **1.7** | | **3.0** | | **-** | **-** | **1.1** | | **-** | | **-** | |  | |
|  |  | 0.2 | | 0.3 | | - | - | 0.5 | | - | | - | |  | |
| P I | | **-** | | **-** | | **3.7** | **5.2** | **-** | | **4.0** | | **2.4** | |  | |
|  |  | - | | - | | 0.1 | 0.07 | **-** | | 0.005** | | 0.1 | |  | |
| N III | | **-** | | **-** | | **2.07** | **3.0** | **0.4** | | **-** | | **-** | |  | |
|  |  | - | | - | | 0.09 | 0.1 | 0.5 | | **-** | | **-** | |  | |
| P III | | **-** | | **-** | | **-** | **1.4** | **-** | | **1.1** | | **0.7** | |  | |
|  |  | - | | - | | - | 0.7 | **-** | | 0.7 | | 0.3 | |  | |
| S III | | **-** | | **-** | | **-** | **-** | **-** | | **0.8** | | **0.5** | |  | |
|  |  | - | | - | | - | - | - | | 0.5 | | 0.3 | |  | |
| N IV | | **-** | | **-** | | **-** | **-** | **-** | | **6.5** | | **3.9** | |  | |
|  |  | - | | - | | - | - | - | | 0.01* | | 0.02* | |  | |
| P IV | | **-** | | **-** | | **-** | **-** | **-** | | **-** | | **0.6** | |  | |
|  |  | - | | - | | - | - | - | | - | | 0.2 | |  | |
| D. uPARAP  **Median fold**  P value | | **P I** | | **N III** | | **P III** | **S III** | **N IV** | | **P IV** | | **S IV** | |  | |
| N I | | **1.1** | | **0.8** | | **-** | **-** | **1.2** | | **-** | | **-** | |  | |
|  |  | 0.8 | | 0.6 | | - | - | 0.3 | | - | | - | |  | |
| P I | | **-** | | **-** | | **0.9** | **0.7** | **-** | | **1.8** | | **1.8** | |  | |
|  |  | - | | - | | 0.7 | 0.7 | **-** | | 0.05 | | 0.1 | |  | |
| N III | | **-** | | **-** | | **1.2** | **1.0** | **1.6** | | **-** | | **-** | |  | |
|  |  | - | | - | | 0.9 | 0.8 | 0.4 | | **-** | | **-** | |  | |
| P III | | **-** | | **-** | | **-** | **0.8** | **-** | | **2.0** | | **2.0** | |  | |
|  |  | - | | - | | - | 0.9 | **-** | | 0.2 | | 0.3 | |  | |
| S III | | **-** | | **-** | | **-** | **-** | **-** | | **2.4** | | **2.4** | |  | |
|  |  | - | | - | | - | - | - | | 0.3 | | 0.3 | |  | |
| N IV | | **-** | | **-** | | **-** | **-** | **-** | | **1.6** | | **1.6** | |  | |
|  |  | - | | - | | - | - | - | | 0.1 | | 0.3 | |  | |
| P IV | | **-** | | **-** | | **-** | **-** | **-** | | **-** | | **1.0** | |  | |
|  |  | - | | - | | - | - | - | | - | | 0.9 | |  | |
| E. EDA **median** fold  P value | | **P I** | | **N III** | | **P III** | **S III** | **N IV** | | **P IV** | | **S IV** | |  | |
| N I | | **1.0** | | **0.9** | | **-** | **-** | **3.1** | | **-** | | **-** | |  | |
|  |  | 0.3 | | 0.5 | | - | - | 0.1 | | - | | - | |  | |
| P I | | **-** | | **-** | | **1.3** | **1.4** | **-** | | **4.0** | | **3.8** | |  | |
|  |  | - | | - | | 0.7 | 0.9 | **-** | | 0.2 | | 0.2 | |  | |
| N III | | **-** | | **-** | | **1.4** | **1.5** | **3.2** | | **-** | | **-** | |  | |
|  |  | - | | - | | 0.5 | 0.8 | 0.6 | | **-** | | **-** | |  | |
| P III | | **-** | | **-** | | **-** | **1.1** | **-** | | **3.0** | | **2.9** | |  | |
|  |  | - | | - | | - | 0.8 | **-** | | 0.5 | | 0.7 | |  | |
| S III | | **-** | | **-** | | **-** | **-** | **-** | | **2.8** | | **2.6** | |  | |
|  |  | - | | - | | - | - | - | | 0.3 | | 0.4 | |  | |
| N IV | | **-** | | **-** | | **-** | **-** | **-** | | **1.3** | | **1.3** | |  | |
|  |  | - | | - | | - | - | - | | 0.4 | | 0.4 | |  | |
| P IV | | **-** | | **-** | | **-** | **-** | **-** | | **-** | | **0.9** | |  | |
|  |  | - | | - | | - | - | - | | - | | 0.9 | |  | |

Median calculated optical densities normalized to GAPDH values are shown in A, while B-E correspond to the indicated **median fold differences** and corresponding P values obtained using the Mann-Whitney test. Relative P value significances were designated as extremely***, very**, or significant*. Roman numbers (I, III and IV) correspond to the original collaborative tumor stages from where fibroblasts were harvested. The tissue sources rendering the fibroblasts used in the study are marked as **N** for normal kidney, **P** for primary RCC and **S** for secondary (metastatic) RCC.
