# Supplementary material for: Elevated Expression of Stromal Palladin Predicts Poor Clinical Outcome in Renal Cell Carcinoma
Source: PLoS One. 2011 Jun 28;6(6):e21494. doi: 10.1371/journal.pone.0021494 (PMC3125241; doi:10.1371/journal.pone.0021494)
Supplement: Table S4 — Expression levels of in vivo stroma markers α-SMA and palladin analyzed by immunohistochemistry. Table listing collaborative stages where * corresponds to samples used for in vitro analyses while a, b and c serve to differentiate among the cases as in Table 1. Types of tissues used are depicted as normal, as well as primary or secondary for tumors. Blinded assessment of immunohistochemistry expression levels using -, -/+, +, ++ and +++ as scoring method. The blinded individual explained the observed stroma (as opposed to epithelial) positive staining of all samples under “Description of stromal expression.” (DOC) [file pone.0021494.s004.doc]

**Table S4**: Expression levels of *in vivo* stroma markers α-SMA and palladin analyzed by immunohistochemistry.

| **Stage** | **Tissue** | **α-SMA** | **Description of stromal expression** |
| --- | --- | --- | --- |
| I* | Primary | + | Well-distinguished mesh |
| III a* | Normal | – | Some + areas around tubules |
|  | Primary | ++ | Fibroblastic proliferation |
| III b* | Primary | + | Mesh (not as delicate) |
| III | Secondary | +/– | Associated with blood vessels |
| IV a* | Normal | – | Diffuse staining around tubule |
|  | Primary | + | Within tumor, increased desmoplasia and fibrosis |
| IV b* | Normal | +/– | Some areas show diffuse staining around tubule |
|  | Primary | +++ | Stained in tumor stroma |
| IV c* | Normal | +/– | Some places is strong |
|  | Primary | + | Cells fibroblast-type are well stained |
| IV | Secondary | ++ | Fine mesh not associated with blood vessels |
| **Stage** | **Tissue** | **palladin** | **Description of stromal expression** |
| I* | Primary | + | Tumor stroma stained positive |
| III a* | Normal | –/+ | Tubules stain positive |
|  | Primary | ++ | Stroma around tumor stain positive |
| III b* | Normal | – | Tubules stain positive |
|  | Primary | + | Tumor stroma stain positive |
| III | Secondary | + | Some staining in tumor stroma |
| IV a* | Secondary | + | Some stromal cells around tumor stain positive |
| IV b* | Normal | – | Diffuse staining |
|  | Primary | + | Stroma stain positive |
| IV c* | Normal | – | No stroma staining was evident |
|  | Primary | –/+ | Some staining in tumor stroma |

Table listing collaborative stages where * corresponds to samples used for *in vitro* analyses while **a**, **b** and **c** serve to differentiate among the cases as in Table 1. Types of tissues used are depicted as normal, as well as primary or secondary for tumors. Blinded assessment of immunohistochemistry expression levels using -, -/+, +, ++ and +++ as scoring method. The blinded individual explained the observed stroma (as opposed to epithelial) positive staining of all samples under “Description of stromal expression.”
